# Supplementary material for: A Predictive Structural Model of the Primate Connectome
Source: Sci Rep. 2017 Mar 3;7:43176. doi: 10.1038/srep43176 (PMC5335700; doi:10.1038/srep43176)
Supplement: Supplementary Figures and Tables [file srep43176-s1.doc]

# A Predictive Structural Model of the Primate Connectome

**Sarah F. Beul^1^, Helen Barbas^2,3^, Claus C. Hilgetag^1,2^**

^1^ Department of Computational Neuroscience, University Medical Center Hamburg-Eppendorf, Martinistr. 52, 20246 Hamburg, Germany

^2^ Neural Systems Laboratory, Department of Health Sciences, Boston University, 635 Commonwealth Ave., 02215 Boston, MA, USA

^3^ Boston University School of Medicine, Department of Anatomy and Neurobiology, 72 East Concord St., 02118 Boston, MA, USA

**Supplementary Data**

**
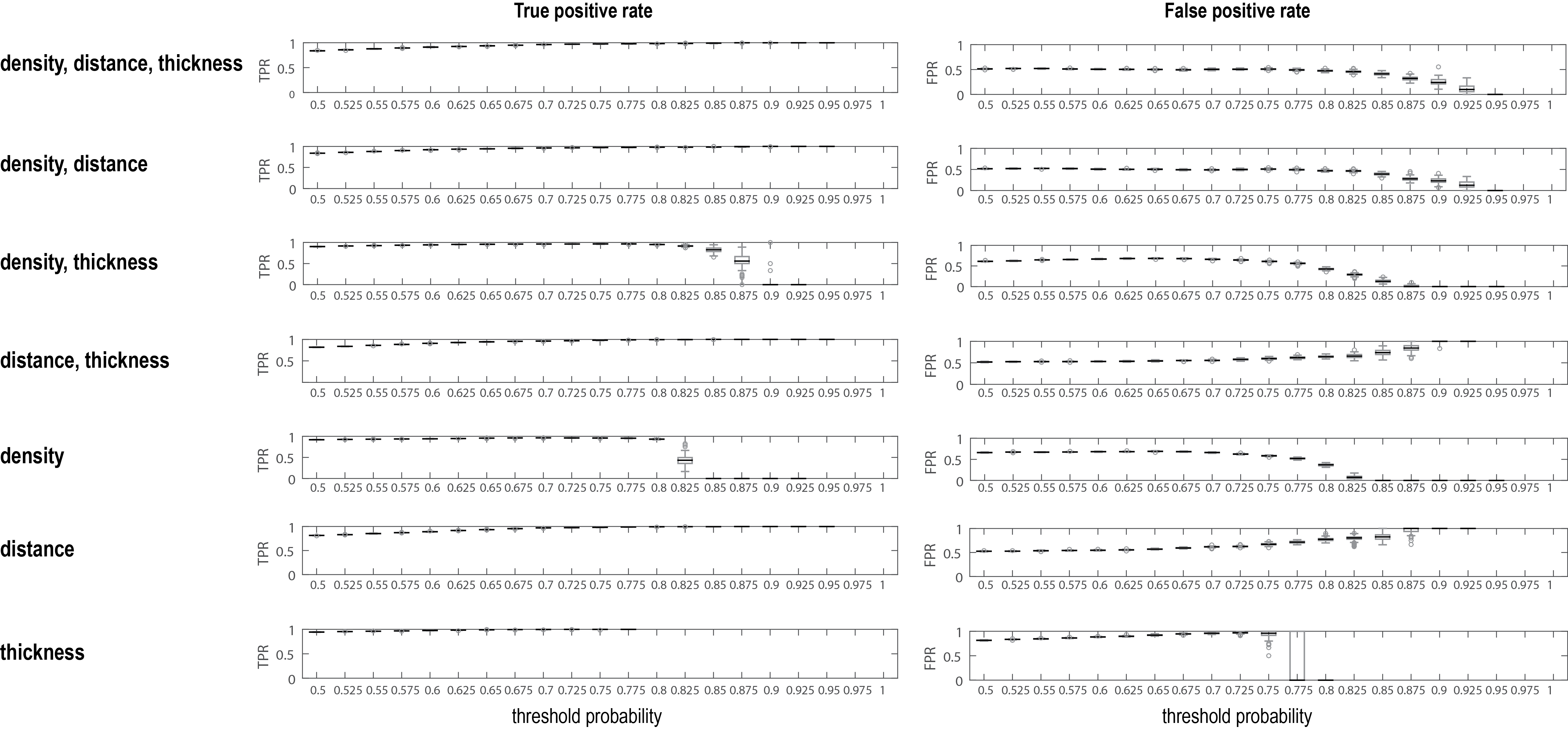
**

**Supplementary Figure S1:** True positive rate and false positive rate for classification of projection existence from all possible combinations of parameters. Distribution of rates across all 100 rounds of cross-validation is shown for all threshold probabilities. Overall performance was best for the combination of |log-ratio_density_| and Euclidean distance. Note that the addition of |log-ratio_thickness_| to these two parameters did not improve performance. Boxplots indicate median rates by a black bar and outliers by gray circles.


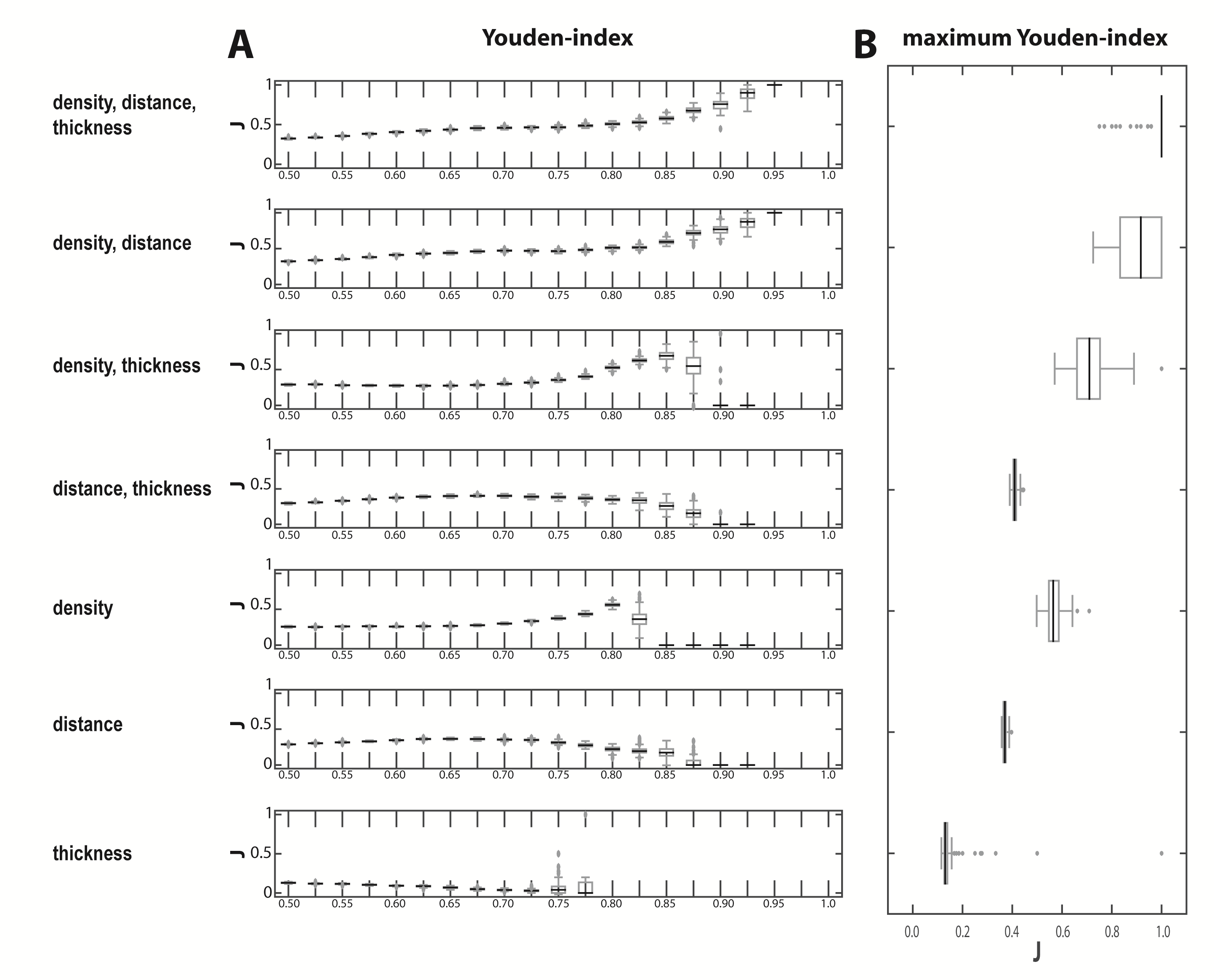


**Supplementary Figure S2:** Youden-index *J* for classification of projection existence from all possible combinations of parameters. (A) Distribution of *J* across all 100 rounds of cross-validation is shown for all threshold probabilities. Overall performance was best for the combination of |log-ratio_density_| and Euclidean distance. Note that the addition of |log-ratio_thickness_| to these two parameters did not improve performance. (B) Distribution of maximum *J* (across all threshold probabilities) for all 100 rounds of cross-validation. Kruskal-Wallis-test showed that the distributions were significantly different (*H* = 661.0, *p* < .001). *Post hoc* tests (Bonferroni-corrected) revealed that the distributions of ‘density, distance, thickness’ and ‘density, distance’ were not significantly different from each other (*p* > .05), while all other pair-wise tests reached statistical significance (all *p* < .001). Boxplots indicate median *J* by a black bar and outliers by gray circles.

**Supplementary Table S1, related to Figure 3**: Classification of unsampled projections. The status of projections not sampled in the data set was predicted from the posterior probabilities resulting from the trained classifier (Fig. 3). Projections were predicted to be absent if their associated |log-ratio_density_| and Euclidean distance yielded a posterior probability for a projection to be present of *p(present)* <= 0.15, and predicted to be present if *p(present)* >= 0.85.

| **Projections predicted to be absent** | |
| --- | --- |
| **Source area** | **Target area** |
| V1 | 9 |
| V2 | 9 |
| V1 | 11 |
| V1 | 12 |
| V1 | 13 |
| V1 | 14 |
| V1 | 25 |
| V1 | 32 |
| V2 | 32 |
| V1 | 24a |
| V1 | 24d |
| V1 | 46v |
| V1 | 8r |
| V1 | F3 |
| V1 | F4 |
| V1 | F6 |
| V1 | OPAI |
| V1 | OPRO |
|  |  |
| **Projections predicted to be present** | |
| **Source area** | **Target area** |
| 10 | 9 |
| 11 | 9 |
| 12 | 9 |
| 13 | 9 |
| 14 | 9 |
| 25 | 9 |
| 32 | 9 |
| 24a | 9 |
| 24c | 9 |
| 24d | 9 |
| 46d | 9 |
| 46v | 9 |
| 8b | 9 |
| 8m | 9 |
| 8r | 9 |
| 9/46d | 9 |
| **Source area** | **Target area** |
| 9/46v | 9 |
| F2 | 9 |
| F3 | 9 |
| F6 | 9 |
| F7 | 9 |
| 9 | 11 |
| 10 | 11 |
| 12 | 11 |
| 13 | 11 |
| 14 | 11 |
| 25 | 11 |
| 32 | 11 |
| 24a | 11 |
| 24c | 11 |
| 24d | 11 |
| 46d | 11 |
| 46v | 11 |
| 8b | 11 |
| 8l | 11 |
| 8m | 11 |
| 8r | 11 |
| 9/46d | 11 |
| 9/46v | 11 |
| F6 | 11 |
| F7 | 11 |
| 9 | 12 |
| 10 | 12 |
| 11 | 12 |
| 13 | 12 |
| 14 | 12 |
| 25 | 12 |
| 32 | 12 |
| 24a | 12 |
| 24c | 12 |
| 24d | 12 |
| 46d | 12 |
| 46v | 12 |
| 8b | 12 |
| 8l | 12 |
| 8m | 12 |
| 8r | 12 |
| 9/46d | 12 |
| 9/46v | 12 |
| F5 | 12 |
| **Source area** | **Target area** |
| F6 | 12 |
| F7 | 12 |
| OPAl | 12 |
| OPRO | 12 |
| 9 | 13 |
| 10 | 13 |
| 11 | 13 |
| 12 | 13 |
| 14 | 13 |
| 25 | 13 |
| 32 | 13 |
| 24a | 13 |
| 24c | 13 |
| 24d | 13 |
| 46d | 13 |
| 46v | 13 |
| 8b | 13 |
| 8l | 13 |
| 8m | 13 |
| 8r | 13 |
| 9/46d | 13 |
| 9/46v | 13 |
| F3 | 13 |
| F4 | 13 |
| F5 | 13 |
| F6 | 13 |
| F7 | 13 |
| OPAl | 13 |
| OPRO | 13 |
| 9 | 14 |
| 10 | 14 |
| 11 | 14 |
| 12 | 14 |
| 13 | 14 |
| 25 | 14 |
| 32 | 14 |
| 24a | 14 |
| 24c | 14 |
| 24d | 14 |
| 46d | 14 |
| 46v | 14 |
| 8r | 14 |
| 9/46d | 14 |
| 9/46v | 14 |
| **Source area** | **Target area** |
| F6 | 14 |
| OPAl | 14 |
| 24d | 23 |
| 8b | 23 |
| 8l | 23 |
| 8m | 23 |
| F1 | 23 |
| F2 | 23 |
| F3 | 23 |
| F4 | 23 |
| 9 | 25 |
| 10 | 25 |
| 11 | 25 |
| 12 | 25 |
| 13 | 25 |
| 14 | 25 |
| 32 | 25 |
| 24a | 25 |
| 24c | 25 |
| 24d | 25 |
| 46d | 25 |
| 46v | 25 |
| 8b | 25 |
| 8m | 25 |
| 9/46d | 25 |
| 9/46v | 25 |
| F3 | 25 |
| F6 | 25 |
| OPAl | 25 |
| OPRO | 25 |
| 9 | 32 |
| 10 | 32 |
| 11 | 32 |
| 12 | 32 |
| 13 | 32 |
| 14 | 32 |
| 25 | 32 |
| 24a | 32 |
| 24c | 32 |
| 24d | 32 |
| 46d | 32 |
| 46v | 32 |
| 8b | 32 |
| 8m | 32 |
| **Source area** | **Target area** |
| 9/46d | 32 |
| F3 | 32 |
| F4 | 32 |
| F6 | 32 |
| F7 | 32 |
| OPAl | 32 |
| OPRO | 32 |
| 9 | 24a |
| 11 | 24a |
| 12 | 24a |
| 13 | 24a |
| 14 | 24a |
| 25 | 24a |
| 32 | 24a |
| 24c | 24a |
| 24d | 24a |
| 46d | 24a |
| 46v | 24a |
| 8b | 24a |
| 8m | 24a |
| 9/46d | 24a |
| F2 | 24a |
| F3 | 24a |
| F4 | 24a |
| F6 | 24a |
| F7 | 24a |
| OPAl | 24a |
| OPRO | 24a |
| 9 | 24d |
| 11 | 24d |
| 12 | 24d |
| 13 | 24d |
| 14 | 24d |
| 23 | 24d |
| 25 | 24d |
| 32 | 24d |
| 24a | 24d |
| 24c | 24d |
| 46d | 24d |
| 46v | 24d |
| 8b | 24d |
| 8l | 24d |
| 8m | 24d |
| 8r | 24d |
| **Source area** | **Target area** |
| 9/46d | 24d |
| 9/46v | 24d |
| F2 | 24d |
| F3 | 24d |
| F4 | 24d |
| F6 | 24d |
| F7 | 24d |
| 9 | 46v |
| 10 | 46v |
| 11 | 46v |
| 12 | 46v |
| 13 | 46v |
| 14 | 46v |
| 25 | 46v |
| 32 | 46v |
| 24a | 46v |
| 24c | 46v |
| 24d | 46v |
| 46d | 46v |
| 8b | 46v |
| 8l | 46v |
| 8m | 46v |
| 8r | 46v |
| 9/46d | 46v |
| 9/46v | 46v |
| F3 | 46v |
| F6 | 46v |
| F7 | 46v |
| 2 | 8r |
| 9 | 8r |
| 11 | 8r |
| 12 | 8r |
| 13 | 8r |
| 14 | 8r |
| 24c | 8r |
| 24d | 8r |
| 46d | 8r |
| 46v | 8r |
| 8b | 8r |
| 8l | 8r |
| 8m | 8r |
| 9/46d | 8r |
| 9/46v | 8r |
| F2 | 8r |
| F3 | 8r |
| F4 | 8r |
| F5 | 8r |
| F6 | 8r |
| F7 | 8r |
| OPRO | ento |
| **Source area** | **Target area** |
| peri | ento |
| TEad | ento |
| TEav | ento |
| TH/TF | ento |
| 9 | F3 |
| 13 | F3 |
| 23 | F3 |
| 25 | F3 |
| 32 | F3 |
| 24a | F3 |
| 24c | F3 |
| 24d | F3 |
| 46d | F3 |
| 46v | F3 |
| 8b | F3 |
| 8l | F3 |
| 8m | F3 |
| 8r | F3 |
| 9/46d | F3 |
| F1 | F3 |
| F2 | F3 |
| F4 | F3 |
| F6 | F3 |
| F7 | F3 |
| 13 | F4 |
| 23 | F4 |
| 32 | F4 |
| 24a | F4 |
| 24d | F4 |
| 8b | F4 |
| 8l | F4 |
| 8m | F4 |
| 8r | F4 |
| 9/46d | F4 |
| F1 | F4 |
| F2 | F4 |
| F3 | F4 |
| F5 | F4 |
| F6 | F4 |
| F7 | F4 |
| OPAl | F4 |
| OPRO | F4 |
| 9 | F6 |
| 10 | F6 |
| 11 | F6 |
| 12 | F6 |
| 13 | F6 |
| 14 | F6 |
| 25 | F6 |
| 32 | F6 |
| **Source area** | **Target area** |
| 24a | F6 |
| 24c | F6 |
| 24d | F6 |
| 46d | F6 |
| 46v | F6 |
| 8b | F6 |
| 8l | F6 |
| 8m | F6 |
| 8r | F6 |
| 9/46d | F6 |
| 9/46v | F6 |
| F2 | F6 |
| F3 | F6 |
| F4 | F6 |
| F7 | F6 |
| 5 | LIP |
| 7a | LIP |
| 7m | LIP |
| DP | LIP |
| STPi | LIP |
| V3a | LIP |
| 12 | OPAl |
| 13 | OPAl |
| 14 | OPAl |
| 25 | OPAl |
| 32 | OPAl |
| 24a | OPAl |
| 8b | OPAl |
| F4 | OPAl |
| OPRO | OPAl |
| 12 | OPRO |
| 13 | OPRO |
| 25 | OPRO |
| 32 | OPRO |
| 24a | OPRO |
| ento | OPRO |
| F4 | OPRO |
| F5 | OPRO |
| OPAl | OPRO |
| peri | OPRO |
| 2 | peri |
| ento | peri |
| F5 | peri |
| OPRO | peri |
| TEad | peri |
| TEav | peri |
| TH/TF | peri |
| ento | TEad |
| peri | TEad |
| TEav | TEad |
| **Source area** | **Target area** |
| TEO | TEad |
| TH/TF | TEad |
| 2 | TEav |
| ento | TEav |
| peri | TEav |
| TEad | TEav |
| teo | TEav |
| TH/TF | TEav |
| ento | TH/TF |
| peri | TH/TF |
| TEad | TH/TF |
| TEav | TH/TF |
| 7m | V3a |
| DP | V3a |
| LIP | V3a |
| MT | V3a |
| V2 | V3a |
| V4 | V3a |
